# Metabolism and secretion of yellow pigment under high glucose stress with *Monascus ruber*

**DOI:** 10.1186/s13568-017-0382-5

**Published:** 2017-04-11

**Authors:** Tao Huang, Meihua Wang, Kan Shi, Gong Chen, Xiaofei Tian, Zhenqiang Wu

**Affiliations:** 1 School of Bioscience and Bioengineering, South China University of Technology, Guangzhou, 510006 People’s Republic of China; 2 Dongguan Tianyi Biotech. Co.Ltd., Dongguan, 523000 People’s Republic of China

**Keywords:** *Monascus ruber*, High glucose stress, Pigments secretion, Gene expression, Yellow pigments, Lipids

## Abstract

The biosynthesis of microbial secondary metabolites is induced by a wide range of environmental stresses. In this study, submerged fermentation of *Monascus* yellow pigments by *Monascus ruber* CGMCC 10910 under high glucose stress was investigated. The increase of lipid content was the major contributor to the increase of dry cell weight (DCW), and the lipid-free DCW was only slightly changed under high glucose stress, which benefited the accumulation of intracellular hydrophobic pigments. The fatty acid composition analysis in *Monascus* cell membranes showed that high glucose stress significantly increased the ratio of unsaturated/saturated fatty acid and the index of unsaturated fatty acid (IUFA) value, which would improve the fluidity and permeability of the cell membrane. As a consequence, high glucose stress increased extracellular yellow pigments production by enhancing secretion and trans-membrane conversion of intracellular pigments to the broth. The total yield of extracellular and intracellular yellow pigments per unit of lipid-free DCW increased by 94.86 and 26.31% under high glucose stress compared to conventional fermentation, respectively. A real-time quantitative PCR analysis revealed that the expression of the pigment biosynthetic gene cluster was up-regulated under high glucose stress. The gene *mppE*, which is associated with yellow pigment biosynthesis, was significantly up-regulated. These results  indicated that high glucose stress can shift the *Monascus* pigment biosynthesis pathway to accumulate yellow pigments and lead to a high yield of both extracellular and intracellular yellow pigments. These findings have potential application in commercial *Monascus* yellow pigment production.

**Electronic supplementary material:**

The online version of this article (doi:10.1186/s13568-017-0382-5) contains supplementary material, which is available to authorized users.

## Introduction

*Monascus* pigments are secondary metabolites with polyketide structures that are produced by *Monascus* spp. (Feng et al. 2012), and are usually classified by color (yellow, orange or red) (Patakova 2013). *Monascus* yellow pigments have been widely researched due to their hypolipidemic (Lee et al. 2010), anti-obesity (Lee et al. 2013), anti-inflammation (Hsu et al. 2012), anti-tumor (Su et al. 2005; Lee et al. 2013), anti-diabetic and anti-oxidative stress (Shi et al. 2012), which are related to the molecular structures of yellow pigments (Su et al. 2005).

It has long been known that the biosynthesis of microbial secondary metabolites is induced by stress (Ranby 1978). Under stress inducing conditions, microorganisms shift from producing primary metabolites to secondary ones in order to preserve energy sources and essential metabolites for more favorable growth conditions. For example, high temperature (>45 °C) can increase the production of *Monascus* yellow pigments, and a high concentration of sodium chloride inhibited mycelia growth but caused an increase in the production of *Monascus* red pigments (Babitha et al. 2007). *Klebsiella oxytoca* fermented with a high concentration of molasses exhibited increased production of 2, 3-butanediol (Afschar et al. 1991). Increased production of monacolin K was observed when a high concentration of glycerol was used as the sole carbon source for *Monascus purpureus* fermentation with the agricultural residue bagasse used as an inert carrier (Lu et al. 2013). In past studies of *Monascus* pigment fermentation, research has mainly focused on improving cell densities and pigment production in fed-batch cultures with long incubation times (Krairak et al. 2000; Lee et al. 2013; Chen et al. 2015). In fed-batch fermentation of *Monascus*, compared with low glucose concentration, high glucose concentration had different impact on the production of *Monascus* pigments (Chen and Johns 1994), and the characteristics of pigments were shifted in *Monascus anka* fed-batch culture with high cell densities (Chen et al. 2015). Cell membrane is the first barrier of microorganism coping with environmental stress, not only for the nutrients absorption but also for the extracellular products excretion, the absorption and excretion ability of microorganism cell response to the fluidity and permeability of the cell membrane (Zhang and Cheung 2011). Glutamic acid could promote the monacolin K production by regulating the permeability of *Monascus* mycelium and then the secretion of monacolin K was promoted without feedback inhibition from intracellular product (Zhang et al. 2017). The permeability and fluidity of cell membrane depended on the saturability of the containing fatty acid (Wang et al. 2013). As high carbon source but low oxidoreduction potential (ORP) could benefit the production of extracellular water-soluble yellow pigments with *Monascus ruber* CGMCC 10910 (Wang et al. 2017), multifaceted mechanisms of high glucose stress that had impacted the metabolism and secretion of *Monascus* yellow pigments should be further investigated.

Recently, the biosynthetic gene cluster of azaphilone pigments in the *Monascus pilosus* genome and the functions of some critical genes involved in the pigment biosynthetic pathway were reported (Balakrishnan et al. 2013). In the present study, the effect of high glucose stress on the fermentation characteristics of *M. ruber* CGMCC 10910 was investigated. Cell growth and lipid production were analyzed to investigate the relationship between pigment production and lipid metabolism. The fatty acid composition of *Monascus* cell membrane under high glucose stress was analyzed using GC–MS to study the influence of high glucose stress on the fluidity and permeability of the cell membrane. The expression levels of pigment biosynthetic genes under high glucose stress were measured by real-time quantitative PCR with a simultaneous analysis of extracellular and intracellular pigment compositions. By undertaking these investigations, we hoped that the regulatory mechanisms of pigment metabolism during high glucose stress would be revealed.

## Materials and methods

### Microorganism and culture conditions

All experiments in this study were performed with *M. ruber* CGMCC 10910 (China General Microbiological Culture Collection Center, CGMCC 10910), which was cultivated on PDA medium at 30 °C for 7 days and then stored at 4 °C. The seed medium contained (g/L): glucose, 20; yeast extract, 3; peptone, 10; KH_2_PO_4_, 4; KCl, 0.5; and FeSO_4_·7H_2_O, 0.01. The inoculum was incubated in a 250-mL Erlenmeyer flask containing 50 mL of seed medium at 30 °C and was shaken at 180 rpm for 25 h. The conventional fermentation medium contained (g/L): glucose, 50; (NH_4_)_2_SO_4_, 5; KH_2_PO_4_, 5; MgSO_4_·7H_2_O, 0.5; KCl, 0.5; MnSO_4_·H_2_O, 0.03; ZnSO_4_·7H_2_O, 0.01; and FeSO_4_·7H_2_O, 0.01. Fermentation medium containing a higher initial glucose concentration (up to 200 g/L) was used for glucose concentration stress experiments. The fermentation experiment was conducted at 30 °C with shaking at 180 rpm for 8 days in a 250-mL Erlenmeyer flask containing 25 mL of fermentation media and using 2 mL of inoculum. All experiments were performed in triplicate.

### Measurements of pigment and residual glucose concentration, DCW, lipid weight and lipid-free DCW

After fermentation, the spent medium was vacuum filtered through a 0.8 mm mixed cellulose esters membrane, after which the filtrate was diluted. Extracellular pigment production was assessed using a UV–Visible spectrophotometer (Unico, USA) scanning from 300 to 550 nm at 1-nm intervals (Shi et al. 2015). The absorbance units (AU) at the peak wavelength (350 nm) multiplied by the dilution ratio was used as an index of the extracellular yellow pigments concentration (Wang et al. 2017). The residual glucose was determined by the standard 3,5-dinitrosalicylic acid (DNS) method. The mycelia was washed for three times and then dried to a constant weight at 60 °C to determine biomass (dry cell weight, DCW). Some of those dry mycelia were submitted for estimation of lipid content. Lipid content in DCW was determined following the standard method by Bligh and Dyer (1959) with some modifications: 0.2 g of dry mycelia was re-suspended in 6 mL hydrochloric acid solution (4 mol/L), and then the mixture was heated to 100 °C and incubated for 3 min. After this, the mixture was immediately cooled down to have the intact cell structure broken down. A 12 mL of fresh extraction solution (methanol/chloroform, 1:1 v/v) was added into the cooled mixture and mixed for 30 s. After centrifugation at 5000 rpm for 15 min, the lower (chloroform) phase was collected to a new test tube containing 5 mL of 0.1% NaCl solution. After a centrifugation at 3500 rpm for 5 min, the lower (chloroform) phase was collected and evaporated with flushing nitrogen to get the lipid residual. Then the lipid residual was oven dried at 60 °C to a constant weight to determine the lipid weight. The lipid content was the extracted lipid weight (g) from per 100 g DCW. The lipid-free DCW was calculated by deducing the lipid weight from the total DCW (Wang et al. 2015a).

The intracellular pigment concentration was determined following those procedure as follows: mycelia were washed and re-suspended in 25 mL of acidic aqueous ethanol (70% v/v pH 2 with hydrochloric acid); the mixture was then incubated for 1 h and then passed through filter paper; finally, the filtrate (intracellular extract) was diluted for determining the intracellular pigment concentration. A UV–Visible absorbance spectrum of intracellular pigments was taken from 300 nm to 550 nm at 1-nm intervals, and the absorbance units (AU) at peak wavelengths of 410 and 470 nm multiplied by the dilution ratio were used as indexes of the intracellular yellow and orange pigments concentrations (Shi et al. 2015), respectively.

### Analyses of pigment compositions by HPLC

Analyses of sample compositions were performed using an Alliance e2695 HPLC system (Waters, Milford, CT, USA) equipped with a 2998 Photodiode Array (PDA) detector (Waters, Milford, CT, USA) and a Zorbax Eclipse Plus C18 column (250 × 4.6 mm, 5 μm, Agilent, Palo Alto, CA, USA). The temperature of the column oven was set at 30 °C. A mixture of H_3_PO_4_ solution (pH 2.5, phase A) and acetonitrile (phase B) were used as the mobile phase using the following gradient program: 0 min, 80% A, 20% B; 25 min, 20% A, 80% B; 35 min, 20% A, 80% B; 36 min, 80% A, 20% B; 41 min, 80% A, 20% B. The PDA was set at 200–600 nm, and the flow rate of the mobile phase was 0.8 mL/min.

### Analyses of extracellular pigments by LC–MS

Liquid chromatography–mass spectrometry consisted of a HP1100 HPLC system (Agilent, Palo Alto, CA, USA) and a micro TOF-QII mass spectrometer (Bruker, Rheinstetten, Germany). The C18 column and chromatographic conditions were the same as mentioned above, except for mobile phase A (water, 0.1% formic acid).

### Analysis of cell membrane fatty acid composition by GC–MS

After 8 days of fermentation, mycelia in the fermentation broth were collected. The fatty acid in cell membrane of the mycelia was extracted, purified and methylated according to the method described by Wang et al. (2013). After that, the sample dissolved in the *n*-hexane was collected for GC–MS analysis, using an Agilent 6890 GC (Agilent, Santa Clara, CA, USA) coupled to an Agilent 5973 mass selective detector (MSD) (Agilent, Santa Clara, CA, USA), equipped with a HP-5MS column (5% Phenyl Methyl Silox, 30 m–0.25 mm id 0.25 μm film thickness, Agilent, Santa Clara, CA, USA). The front injection was 250 °C with a split ratio of 70:1. Helium gas (purity of 99.9999%, Foshan, China) was used as the carrier gas at a flow rate of 50 mL/min. The oven temperature program was as follows: 80 °C for 2 min, then raised to 150 °C at a rate of 10 °C/min, and then further to 230 °C at a rate of 3 °C/min, keeping at 230 °C for 5 min. The electron impact energy was 70 eV, and the ion source temperature was set at 230 °C.

### Gene expression analysis

The effects of high glucose stress on the expression of key genes during pigments production were investigated using real-time quantitative PCR. Mycelia were collected and stored in liquid N_2_ before total RNA extraction using the Plant RNA Extraction Kit (TakaRa MiniBEST). cDNA was synthesized using the PrimeScript™RT reagent Kit with gDNA Eraser (TaKaRa). Primers for the amplification of *MpFasA2*, *MpFasB2*, *MpPKS5*, *mppR1*, *mppB*, *mppC*, *mppD*, *mppE*, *mppR2* (GenBank accession No. KC148521) and the *actin* gene (GenBank accession No.AJ417880) were listed in Additional file 1: Table S1 according to the previous study (Wang et al. 2015b) with some modifications, *actin* gene was used as a reference gene. Gene expression was monitored by RT-qPCR using the SYBR Premix Ex TaqII (TaKaRa). RT-qPCR was performed using a Lightcycler 96 (Roche, USA) with the following cycling program: pre-incubation at 95 °C for 30 s, followed by a two-step amplification (40 cycles of denaturation at 95 °C for 5 s, and annealing at 60 °C for 30 s) and dissociation curve analyses (at 95 °C for 10 s, annealing at 65 °C for 60 s, then collecting dissociation curves from 65 to 95 °C, with a final incubation at 97 °C for 1 s).

### Statistical analysis

Each experiment was repeated at least in triplicate. Numerical data are presented as the mean ± SD. The differences among different treatments were analyzed using one-way ANOVA. All statistical analyses were performed by using SPSS 22.0, software. *p* < 0.05, *p* < 0.01 was considered statistically significant.

## Results

### Production of *Monascus* pigments and lipids during high glucose stress fermentation

**Fig. 1:**
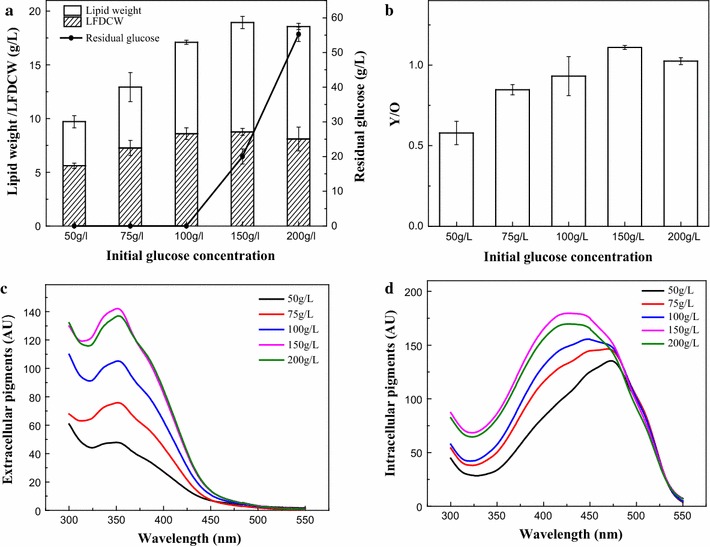
Metabolism of lipid and pigments with IGC of 50, 75, 100, 150 and 200 g/L. **a** Lipid weight (g/L), LFDCW (g/L) and residual glucose (g/L). **b** Ratio of intracellular yellow to orange pigments (Y/O).**c** Spectra of extracellular pigments. **d** Spectra of intracellular pigments

The dry cell weight (DCW) of cells takes into account both the accumulation of lipids and lipid-free dry cell weight (LFDCW) accumulation (Wang et al. 2015a). We observed that the final DCW (the sum of lipid weight and LFDCW) increased with an increase in initial glucose concentration (IGC), and that the majority of this DCW increase was attributable to an increase of lipid weight at an IGC > 100 g/L while LFDCW increased only slightly or even decreased when IGC was up to 200 g/L (Fig. 1a). Extracellular yellow pigments production increased sharply with an increased IGC and reached approximately 147 AU_350_ at 150 g/L IGC (Fig. 1c), which was approximately twofold higher than when a 50 g/L IGC was used. These pigments were mainly water-soluble yellow pigments with a maximum absorption peak at 350 nm. Intracellular yellow pigments also increased with an increasing IGC, but the pigment hue depended on IGC. The maximum absorbance of intracellular pigments was 470 nm (dominated by orange pigments) under a low IGC (50 g/L) but at high IGCs (>150 g/L) the maximum absorbance shifted to 410 nm (dominated by yellow pigments) (Fig. 1d). The ratio of yellow to orange pigments (Y/O) increased dramatically with an increasing IGC (Fig. 1b).

**Fig. 2:**
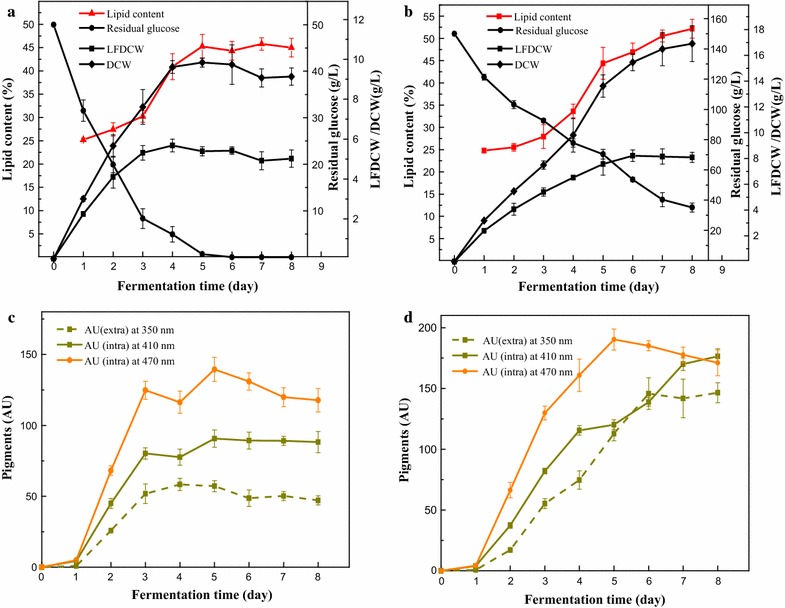
Time course of lipid content (%), LFDCW (g/L), DCW (g/L), pigment production and residual glucose (g/L) under different IGCs. **a**, **c** IGC = 50 g/L. **b**, **d** IGC = 150 g/L

The increase in DCW was mainly attributable to the increased LFDCW during the first 3 days, while the lipid content started to increase rapidly from the 3rd to 5th day at a low IGC of 50 g/L (Fig. 2a). The LFDCW increase extended to the 6th day and the lipid content increased until the 8th day under high glucose stress (Fig. 2b). Extracellular yellow pigments increased with the accumulation of LFDCW and reached a maximum value on the 4th day at which time LFDCW was highest at a low IGC of 50 g/L (Fig. 2c). However, during fermentation with high glucose concentrations, extracellular pigments reached maximum productivity on the 6th day when LFDCW was highest (Fig. 2d). This indicated that production of extracellular water-soluble yellow pigments was related to the LFDCW. On the other hand, intracellular pigments (yellow and orange) increased with the accumulation of DCW, reaching a maximum value on the 5th day at which time DCW was highest at an IGC of 50 g/L. The orange pigments began to decrease from the 5th day while yellow pigments remained unchanged (Fig. 2c). During high glucose stress, intracellular orange pigments reached the maximum value on the 5th day and then began to decrease while intracellular yellow pigments increased continuously to the 8th day (Fig. 2d). The decrease in pigments during the later stage of fermentation indicated the decomposition or transformation from orange pigments into yellow pigments. The production of intracellular pigments was well correlated to cell growth, including both of LFDCW and lipid weight.

Compared to conventional fermentation with 50 g/L IGC, the maximum total yields of extracellular and intracellular yellow pigments increased by 194 and 101%, respectively, while the total respective yields of extracellular and intracellular yellow pigments per unit LFDCW improved by 94.86 and 26.31% and intracellular orange pigments decreased by 10.85% (Table 1) under high glucose stress (IGC = 150 g/L). Those results demonstrated that a high concentration of glucose benefited the production of yellow pigments, which was due to an increase of DCW and biosynthetic capacity of pigments.

**Table 1:** Pigment yield per unit LFDCW and yield increase rate under high glucose stress

Pigments	Yield (AU per g LFDCW)^b^		
	50 g/L^a^	150 g/L^a^	Increase rate (%)^c^
Extracellular yellow	9.326 ± 0.054	18.173 ± 0.050	94.86
Intracellular yellow	17.376 ± 0.188	21.947 ± 0.111	26.31
Intracellular orange	23.688 ± 0.246	21.118 ± 0.049	−10.85

^a^Initial glucose concentration

^b^Pigments yield per unit LFDCW

^c^Yield increase rate at 150 g/L IGC compared to 50 g/L IGC

**Fig. 3:**
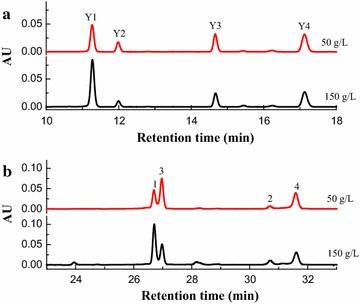
HPLC-PDA chromatogram of pigments fermented under different IGCs. **a** Extracellular pigments. **b** Intracellular pigments. Y1, Y2, Y3 and Y4 are four extracellular water-soluble yellow pigments. *1* monascin, *2* ankaflavin, *3* rubropunctation, *4* monascorubrin

It was worthy to note that four extracellular water-soluble yellow pigments (**Y1**–**Y4**) were found in the spent broth (Fig. 3a). **Y1** had the UV–Visible spectra with two maximum absorptions at around 225 nm and 337 nm, **Y2** had the UV–Visible spectra with two maximum absorptions at around 215 nm and 361 nm, **Y3** and **Y4** had almost the same UV–Visible spectra with two maximum absorption at around 218, 291 and 388 nm (Additional file 2: Figure S1). The extracellular broth gave rise to a comprehensive absorption peak at 350 nm. The intracellular pigments were mainly composed of four well-known pigments, including two yellow pigments (monascin and ankaflavin) and two orange pigments (monascorubrin and rubropunctation) but no red pigments (Fig. 3b; Additional file 3: Figure S2). This may have been caused by the use of ammonium sulfate as a nitrogen source that led to a low pH (<2.5) of the broth, which was good for the accumulation of yellow and orange pigments (Shi et al. 2015). In our study, ammonium sulfate used as a sole nitrogen source, resulted in a very low pH below 2.0. Interestingly, the ratio of intracellular yellow pigments (monascin and ankaflavin) to orange pigments (rubropunctation and monascorubrin) increased under high glucose stress. Under the high glucose stress, yields of the intracellular yellow pigments monascin and ankaflavin, respectively, increased by 94.6 and 51.4% based on peak areas compared to when a low IGC of 50 g/L was used. These results demonstrated that high glucose stress could result in a high proportion of yellow pigments during *Monascus* cultivation.

### Changes of fatty acids composition in cell membrane

The major fatty acid components in the membrane of *M. ruber* CGMCC 10910 were identified as tetradecanoic acid (C14:0), palmitic acid (C16:0), stearic acid (C18:0), eicosanoic acid (20:0), oleic acid (C18:1), linoleic acid (C18:2) and linolenic acid (C18:3), respectively (Table 2). It could be found that the saturated fatty acids, especially stearic acid (C18:0), had a significant decreased but the unsaturated fatty acids, especially oleic acid (C18:1), increased under the high glucose stress (150 g/L). The unsaturated/saturated fatty acid ratio and the index of unsaturated fatty acid (IUFA) value increased significantly from 1.520 to 2.028 and from 79.295 to 89.055, respectively. It was suggested that the *M. ruber* CGMCC 10910 would synthesize more unsaturated fatty acids under high glucose stress which could improve the fluidity and permeability of the cell membrane (Zhang and Cheung 2011; Lyu et al. 2015), and then facilitate trans-membrane secretion and conversion of intracellular pigments to the broth (Chen et al. 2017).

**Table 2:** Fatty acid composition (% total fatty acid) of cell membranes under high glucose stress

Fatty acid composition		IGC (g/L)	
		50	150
Saturated fatty acid	Tetradecanoic acid (14:0)	0.123 ± 0.01	0.157 ± 0.02
	Palmitic acid (16:0)	14.641 ± 0.22	14.431 ± 0.18
	Stearic acid (18:0)	23.978 ± 0.56	17.862 ± 0.47
	Eicosanoic acid (20:0)	0.293 ± 0.03	0.170 ± 0.01
Unsaturated fatty acid	Palmitoleic acid (16:1)	0.161 ± 0.02	0.395 ± 0.05
	Oleic acid (18:1)	40.649 ± 0.86	45.596 ± 0.78
	Linoleic acid (18:2)	17.058 ± 0.35	17.439 ± 0.27
	Linolenic acid (18:3)	1.457 ± 0.08	2.730 ± 0.12
Unsaturated/saturated^a^		1.520 ± 0.01 a	2.028 ± 05 A
IUFA (index of unsaturated fatty acid)^b^		79.295 ± 0.68 b	89.055 ± 0.54 B

Data are mean ± standard deviation (n = 3). Means in a row with different lowercase/capital letters are significantly different (*p* < 0.05)

^a^(C18:1 + C18:2 + C18:3)/(C14:0 + C16:0 + C17:0 + C18:0 + C20:0)

^b^(C18:1) + (C18:2) × 2 + (C18:3) × 3

### Expression levels of pigment biosynthetic genes

**Fig. 4:**
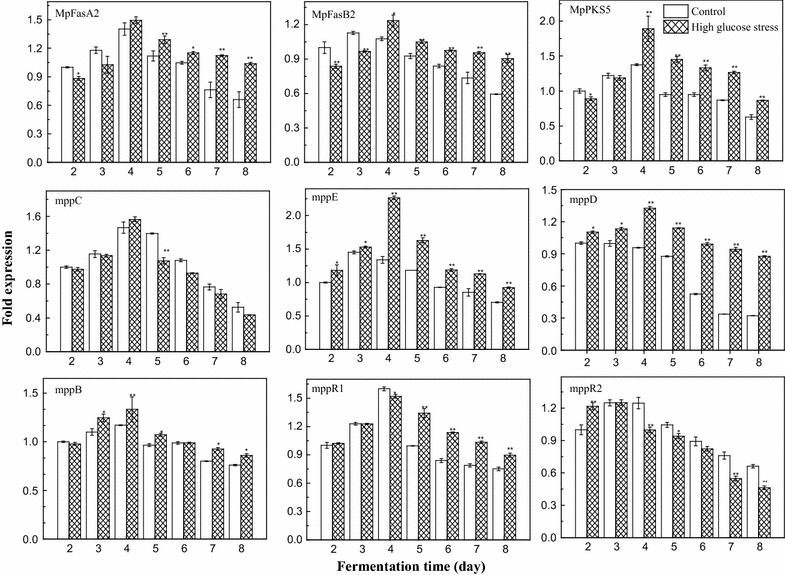
Relative expression levels of the pigment biosynthesis related genes *MpFasA2, MpFasB2, MpPKS5, mppB, mppC, mppD, mppE, mppR1* and *mppR2* as monitored by qRT-PCR. *Error bars* represent the standard deviation (n = 3). **p < 0.01, *p < 0.05

The expression levels of the pigment biosynthetic genes *MpFasA2, MpFasB2, MpPKS5, mppB, mppC, mppD, mppE, mppR1* and *mppR2* during the fermentation course under high glucose stress (IGC = 150 g/L) were monitored by RT-qPCR (Fig. 4). Gene expression test samples corresponded one-to-one with the samples used for pigments testing. Transcriptional levels were normalized to that of the *actin* gene. To standardize the results, we took the mRNA levels accumulated during the 2nd day of the control (IGC = 50 g/L) as the reference value (value 1). The expression levels of the pigment biosynthetic genes first increased, and then decreased during the fermentation under high glucose stress. During the first 3 days, the expression levels of the genes *mppE, mppD* and regulatory gene *mppR2* were significantly up-regulated under the high glucose stress. In the middle and later stages of the fermentation (from the 3rd day to the 8th day), the expression levels of the genes *MpFasA2, MpFasB2, MpPKS5, mppB, mppD, mppE,* and *mppR1* were significantly up-regulated (*p* < 0.01 or *p* < 0.05) and they were all higher than the control. But the expression levels of the gene *mppC* and the regulatory gene *mppR2* were down-regulated. These results demonstrated that high glucose stress could regulate gene expression for pigment biosynthesis, and increase production of both intracellular and extracellular pigments (Fig. 1).

During the fermentation anaphase (after the 6th day), the expression levels of *MpFasA2, MpFasB2, MpPKS5, mppD, mmpB,* and *mppR1* were significantly up-regulated (*p* < 0.01 or *p* < 0.05). As the genes *MpFasA2, MpFasB2, MpPKS5, mppD*, and *mppB* are structural genes for pigment biosynthesis and *mppR1* is a regulatory gene (Balakrishnan et al. 2013), the polyketide chromophores and media fatty acid were still being generated during fermentation anaphase under high glucose stress. Simultaneously, the gene *mppE* for yellow pigment biosynthesis (Balakrishnan et al. 2017) was significantly up-regulated, while the gene *mppC* for orange pigment biosynthesis (Liu et al. 2014) was down-regulated in some degree. In combination with the time course of pigment production, the up-regulation of *mppE* was positively correlated with the production of yellow pigments in the later stages of fermentation.

## Discussion

*Monascus* pigments are mixtures with multi-components (Juzlova et al. 1996; Patakova 2013). The concentration of *Monascus* pigments is usually represented by the absorbance at their characteristic wavelength (Babitha et al. 2007). Thus, the pigments yield in this study was represented by the absorbance at their characteristic wavelength (350, 410, and 470 nm). Submerged fermentation of *Monascus* species with a low IGC in the medium resulted in the accumulation of intracellular orange *Monascus* pigments exhibiting a peak at 470 nm (Kang et al. 2014). In this study, high yields of both extracellular and intracellular yellow pigments were obtained using *M. ruber* CGMCC 10910 when the IGC were increased from 50 g/L (low) to >150 g/L (high). An interesting phenomenon was observed that the dominating intracellular pigments changed from orange to yellow pigments (Fig. 1). In the later stage of fermentation under high glucose stress, the accumulation of DCW was mostly attributable to the increased intracellular lipid weight as the LFDCW was only slightly changed when the IGC was higher than 100 g/L. When the IGC was 150 g/L, the lipid weight reached approximately 53% of the DCW, 20% higher than what was observed at a low glucose concentration (IGC = 50 g/L). It has been reported that *Monascus purpureus* albino strain accumulated a high content of lipids under a limited nitrogen condition (carbon to nitrogen = 80:1) (Rasheva et al. 1997). The high lipid production observed in this study was also caused by a high ratio of carbon to nitrogen in the media. Lipid droplets in living microorganisms could serve as a reservoir for intracellular *Monascus* pigments, and there was a positive correlation between intracellular pigments and microbial lipids  (Wang et al. 2015a). The intracellular yellow pigments and lipid content all increased continuously to the 8th day under high glucose stress (Fig. 2), the reason was that the intracellular lipids act as reservoirs for intracellular yellow pigments storage. Thus, high glucose stress increased the content of *Monascus* mycelia mainly by increasing the lipids content of *Monascus* mycelia, which can improve more reservoirs for intracellular yellow pigments storage  (Wang et al. 2015a), thus enhancing intracellular yellow pigments production.

Except for extractive fermentation, most of *Monascus* pigment studies focused on the intracellular pigments biosynthesis (Balakrishnan et al. 2013, 2014, 2017; Bijinu et al. 2014), while only a small amount of research had been done on the biosynthesis pathway of extracellular pigments (Koehler 1983; Hajjaj et al. 1997). Hajjaj et al. (1997) discovered that *Monascus* could produce the extracellular red pigments N-glucosylrubropunctamine and N-glucosylmonascorubramine in a chemically defined culture medium with excess glucose and monosodium glutamate (nitrogen source). Chen et al. (2017) found that the intracellular orange pigments could be converted to extracellular yellow pigments during the trans-membrane secretion process in a nonionic surfactant aqueous solution (Chen et al. 2017). So, we speculated that the extracellular water-soluble yellow pigments in this study were derivatives of intracellular pigments via the trans-membrane conversion. The pigments were further identified by means of LC–MS (Additional file 4: Figure S3). Based on their UV–Visible spectra (Additional file 2: Figure S1) and molecular weights, It could be deduced that the four pigments have not been described and reported before (Chen and Wu 2016). It needed to be confirmed by identifying the structure of four extracellular water-soluble yellow pigments further. We could also observe that the production of extracellular water-soluble yellow pigments were growth-associated and were coupled to LFDCW, while the concentration of intracellular pigments was just partially associated with cell growth (Fig. 2). A possible reason for this is that during the earlier stages of fermentation, the increased of DCW was mainly attributable to the increasing LFDCW and lower intracellular lipid accumulated, resulting in fewer reservoirs for intracellular pigment storage. The time accumulated LFDCW was extended under a high IGC (Fig. 2b), which allowed more time for the biosynthesis and secretion of derivative extracellular pigments (water-soluble yellow pigments). During the later stages of the fermentation, the increased DCW was mainly due to increased lipids (Fig. 2b), which may have served as reservoirs for accumulating intracellular pigments and caused less pigments precursors to be available for the conversion and secretion of extracellular water-soluble yellow pigments (Fig. 2d). On the other hand, the high glucose stress could also promote the biosynthesis of unsaturated fatty acids in *M. ruber* and make a better fluidity and permeability of the cell membrane, which would improve the trans-membrane conversion and secretion of intracellular pigments to the broth. The similar report could be found that the fumaric acid production could be improved under high glucose stress through synthesizing more unsaturated fatty acids than the saturated one to alternate the fluidity and permeability of the cell membrane with *Rhizopus oryzae* (Lyu et al. 2015). High glucose stress changed the permeability of *Monascus* mycelia, enhanced the trans-membrane conversion and secretion of intracellular pigments to the broth, and improved the production of extracellular yellow pigments.

The biosynthesis of *Monascus* pigments follows the polyketide pathway (Hajjaj et al. 1997; Shao et al. 2014). *MpPKS5* and *mppD* are the structural genes of *Monascus* pigments and encode the polyketide synthases which are keys to the biosynthesis the polyketide chromophore of these pigments. The genes *MpfasA2* and *MpfasB2* (*Mpfas2*) encode a canonical fungal fatty acid synthase and supply the medium-chain (C8 and C10) fatty acyl moieties for *Monascus* pigments biosynthetic activities (Balakrishnan et al.^2013^, ^2014^). The *mppB* gene encodes a trichothecene 3-O-acetyltransferase (AT), which can transfer the medium-chain (C8 and C10) fatty acyl group into the polyketide chromophore to complete pigment biosynthesis. The *mppR1* and *mppR2* genes are regulatory genes for pigments biosynthesis (Balakrishnan et al. 2013). The genes *MpPKS5*, *MpfasA2*, *MpfasB2*, *mppB*, *mppR1*, and *mppD* were up-regulated during high glucose stress in the later stage of fermentation (Fig. 4). Furthermore, the increased glucose as the sole carbon source could offer more precursors and cofactors such as acetyl-CoA, malonyl-CoA, NADH and NADPH for the biosynthesis of *Monascus* pigments and lipids (Beatriz Ruiz et al. 2010). These results illustrated that the polyketide biosynthesis capacity could be enhanced by increasing the polyketide chromophores, medium-chain fatty acyl moieties and critical polyketide synthases under high glucose stress. It helped support that high glucose stress promoted the production of yellow pigments through an internal power and the promoting effect is stable.

**Fig. 5:**
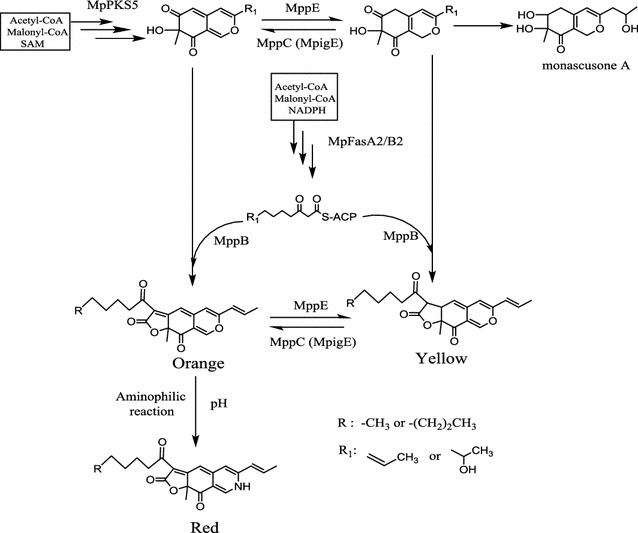
Putative biosynthetic pathway of *Monascus* pigments

The gene *mppE* encodes a reductive enzyme which controls the biosynthesis of the yellow pigments (ankaflavin and monascin) in the polyketide biosynthesis pathway. The production of orange pigment was enhanced, while that of the yellow pigments decreased in an *mppE* knockout mutant (*ΔmppE*). The production of yellow pigments was only enhanced with marked reductions in other pigments in an *mppE* overexpression strain (*OV*-*mppE*) (Balakrishnan et al. 2017). Up-regulation of *mppE* occurred during an increase in yellow pigments (ankaflavin and monascin) and a decrease in orange pigments under blue light stimulation (Chen et al. 2016). The *mppC* gene also encodes an oxidoreductase that shares a 98% consensus of amino acid sequence with MpigE in *Monascus ruber* M7. The *MpigE* deletion strain (*ΔMpigE*) just yielded four kinds of yellow pigments but was very limited in red pigments, whereas production of orange and red *Monascus* pigments was recovered by *MpigE* complementation strain (*ΔMpigE::MpigE*) (Liu et al. 2014). The orange pigments monascorubrin and rubropunctatin could be reduced to the yellow pigments ankaflavin and monascin, respectively (Hajjaj et al. 2000). In this study, high glucose stress up-regulated the relative expression level of the gene *mppE* while down-regulated the gene *mppC* and *mppR2* (Fig. 4), which increased more reductive enzymes involved in yellow pigment biosynthesis (Balakrishnan et al. 2017). In addition, high concentration of glucose could provide high reducing power (NADH or NADPH) (Beatriz Ruiz et al. 2010). As a consequence, the intracellular yellow pigments (monascin and ankaflavin) dramatically increased in the later stages of fermentation while intracellular orange pigments decreased to some degree under high glucose stress (Figs. 2d, 3), and resulted in a high yield of yellow pigments. In light of these results, a putative biosynthetic pathway of *Monascus* pigments was shown in Fig. 5, which includes the chemical modification of orange pigments to generate red ones through an aminophilic reaction between orange *Monascus* pigments and primary amine (Jung et al. 2003; Xiong et al. 2015; Shi et al. 2016). In which there may be some oxidoreduction conversion of the polyketide chromophores between yellow and orange pigments or a direct conversion between yellow and orange pigments. The genes *mppE* and *MpigE* (*mppC*) may all be involved in this conversion (Fig. 5).

In summary, high glucose stress improved more reservoirs for intracellular pigments storage by increasing the content of *Monascus* mycelia and the lipids content in *Monascus* mycelia. Simultaneously, high glucose stress up-regulated the expression of pigment biosynthetic genes, especially the genes involved in yellow pigments biosynthetic. Thereby, a high proportion of intracellular yellow pigments rather than orange pigments were achieved under high glucose stress. High glucose stress also improved the fluidity and permeability of the cell membrane and enhanced the trans-membrane conversion of intracellular pigments to extracellular water-soluble yellow pigments and secretion into the broth, resulted in a twofold increase of extracellular water-soluble yellow pigments compared to low IGC condition. Further studies are needed to elucidate the molecular pathways through which high glucose stress regulates yellow pigments production. Thus, submerged fermentation under high glucose stress has potential application in the production of *Monascus* yellow pigments.

## Additional files

**Additional file 1: Table S1.** Primers used for RT-qPCR analyzing pigment biosynthesis genes.

**Additional file 2: Figure S1.** UV-Visible spectra of extracellular pigments detected by HPLC-PDA.

**Additional file 3: Figure S2.** UV-Visible spectra of intracellular pigments detected by HPLC-PDA.

**Additional file 4: Figure S3.** LC-MS analysis of extracellular pigments. **a** Total ion chromatograms, absorption traces of the pigments. **Y1-Y4**, Mass spectra and their collision-induced fragmented data.

## Abbreviations

HPLC: high performance liquid chromatography
DCW: dry cell weight
LFDCW: lipid-free dry cell weight
RT-qPCR: real-time quantitative PCR
GC–MS: gas chromatograph–mass spectrometer
LC–MS: liquid chromatograph–mass spectrometer
