# Supplementary material for: Metabolism and secretion of yellow pigment under high glucose stress with Monascus ruber
Source: AMB Express. 2017 Apr 11;7:79. doi: 10.1186/s13568-017-0382-5 (PMC5388664; doi:10.1186/s13568-017-0382-5)
Supplement: Supplementary file 1 — Additional file 1: Table S1. Primers used for RT-qPCR analyzing pigment biosynthesis genes. [file 13568_2017_382_MOESM1_ESM.doc]

**Supplementary Table 1**

## **Table S1.** Primers used for RT-qPCR analyzing pigment biosynthesis genes

| Primers name | Primers sequences (5′→3′) |
| --- | --- |
| *actin* F | 5′-TTCGAGACCTTCAACGCCC-3′ |
| *actin* R | 5′-ACCCTCGTAGATGGGAACGA-3′ |
| *MpFasA2* F | 5′-ATGGATCGCCCGATCTTGTC-3′ |
| *MpFasA2* R | 5′-CTTTGTCGAGTCCGCTGGAT-3′ |
| *MpFasB2* F | 5′-CCTCCAGGGATTACAACCCG-3′ |
| *MpFasB2* R | 5′-ATTCAATGCCAGGTGCTCCA -3′ |
| *MpPKS5* F | 5′-TGTCCGACGAGTTTCTGCAA-3 |
| *MPPKS5* R | 5′-TATCAACGCTGCTTGGGCAT-3′ |
| *mppR1* F | 5′-TCTGCAGTATGCCATGTGGG-3′ |
| *mppR1* R | 5′-ATGGCACCGTCACTTAGCTC-3′ |
| *mppR2* F | 5′-ACGAAACCCTCCATGACACC-3′ |
| *mppR2* R | 5′-TGCAGACAGCCTTGTGGTAG-3′ |
| *mppB* F | 5′-CGTCTCGCCCGATAACTTCA-3′ |
| *mppB* R | 5′-TTGACAGACGGGTCGAAGTC -3′ |
| *mppC* F | 5′-CAGTCCTCGTCCCTTCCAGT -3′ |
| *mppC* R | 5′-CCACGGTGAAGGATGTCGAG -3′ |
| *mppD* F | 5′-TCAACACGGGAGATGCTGTC-3′ |
| *mppD* R | 5′-GCCAAAGGACAGGAGCAGAT-3′ |
| *mppE* F | 5′-CTTCCCGATGCCGTTGTGAT-3′ |
| *mppE* R | 5′-CGTCTCGTGGATCATCTCGT-3′ |
