# Supplementary material for: Metabolism and secretion of yellow pigment under high glucose stress with Monascus ruber
Source: AMB Express. 2017 Apr 11;7:79. doi: 10.1186/s13568-017-0382-5 (PMC5388664; doi:10.1186/s13568-017-0382-5)
Supplement: Supplementary file 2 — Additional file 2: Figure S1. UV-Visible spectra of extracellular pigments detected by HPLC-PDA. [file 13568_2017_382_MOESM2_ESM.doc]

**Supplementary Figure 1**

**Fig.S1** UV-Visible spectra of extracellular pigments detected by HPLC-PDA.
