# Supplementary material for: Metabolism and secretion of yellow pigment under high glucose stress with Monascus ruber
Source: AMB Express. 2017 Apr 11;7:79. doi: 10.1186/s13568-017-0382-5 (PMC5388664; doi:10.1186/s13568-017-0382-5)
Supplement: Supplementary file 3 — Additional file 3: Figure S2. UV-Visible spectra of intracellular pigments detected by HPLC-PDA. [file 13568_2017_382_MOESM3_ESM.doc]

**Supplementary Figure 2**

**Fig. S2** UV-Visible spectra of intracellular pigments detected by HPLC-PDA.
