# Supplementary material for: Metabolism and secretion of yellow pigment under high glucose stress with Monascus ruber
Source: AMB Express. 2017 Apr 11;7:79. doi: 10.1186/s13568-017-0382-5 (PMC5388664; doi:10.1186/s13568-017-0382-5)
Supplement: Supplementary file 4 — Additional file 4: Figure S3. LC-MS analysis of extracellular pigments. a Total ion chromatograms, absorption traces of the pigments. Y1-Y4, Mass spectra and their collision-induced fragmented data. [file 13568_2017_382_MOESM4_ESM.doc]

**Supplementary Figure 3**


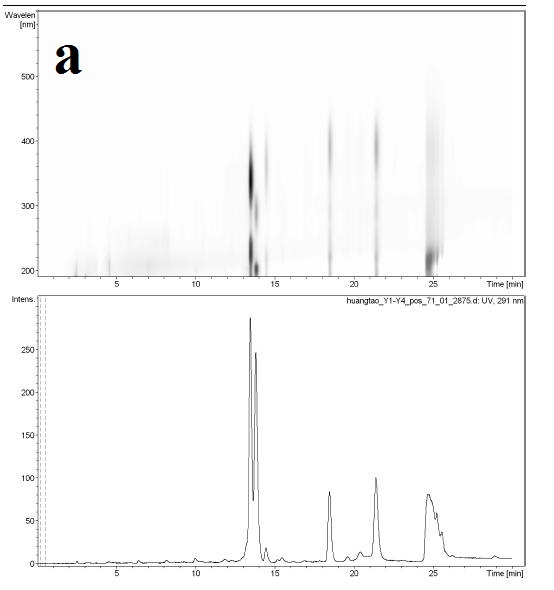


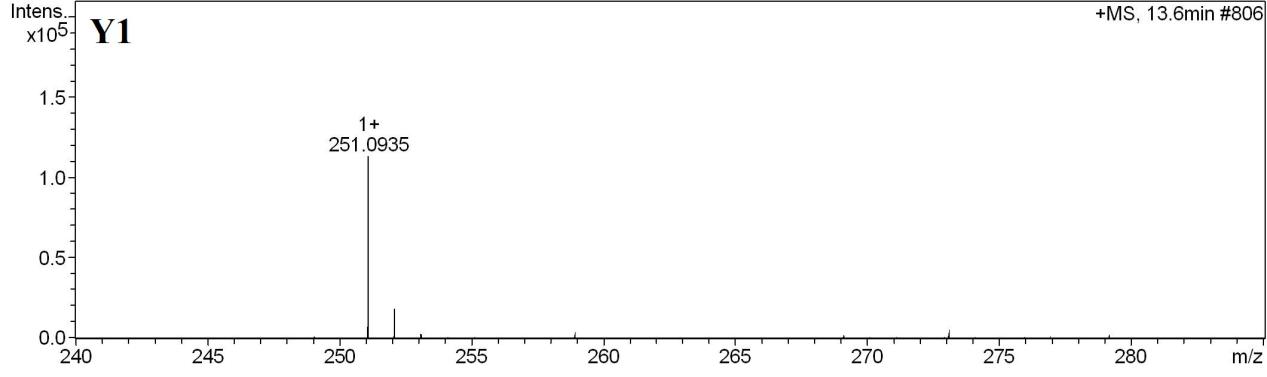

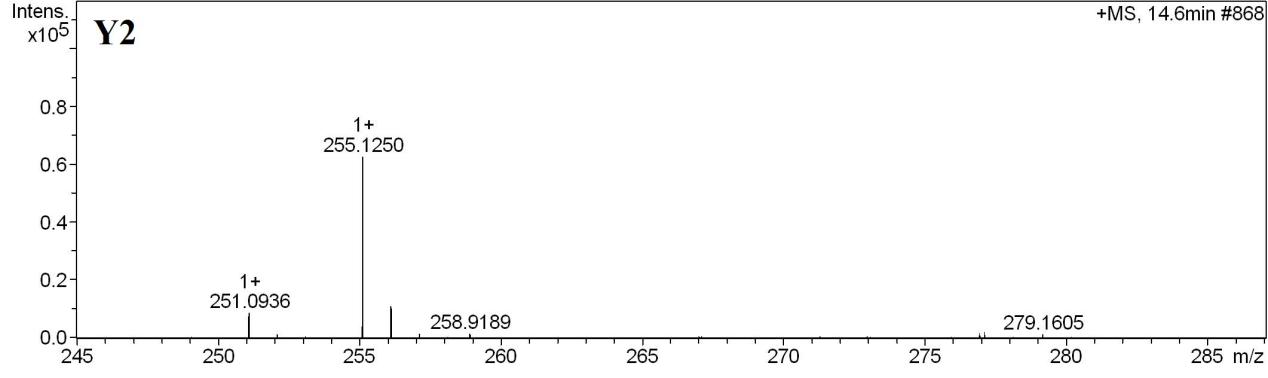

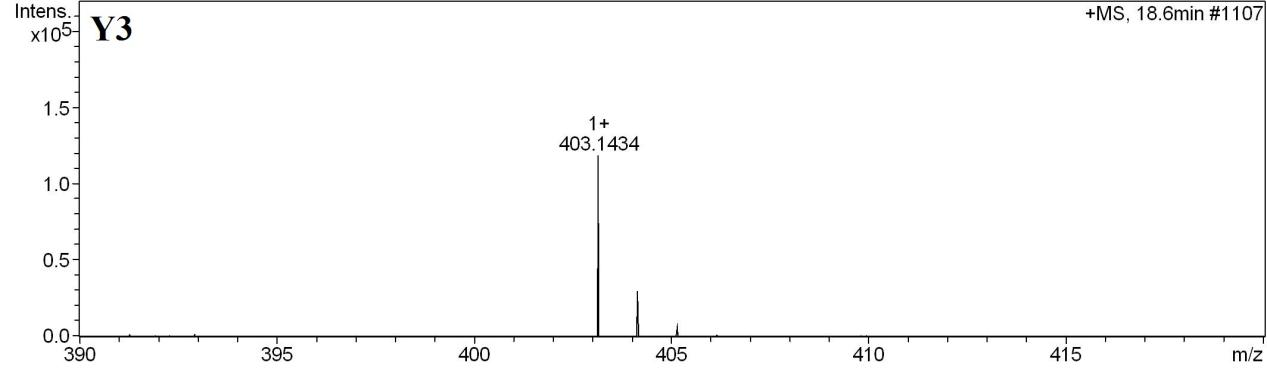

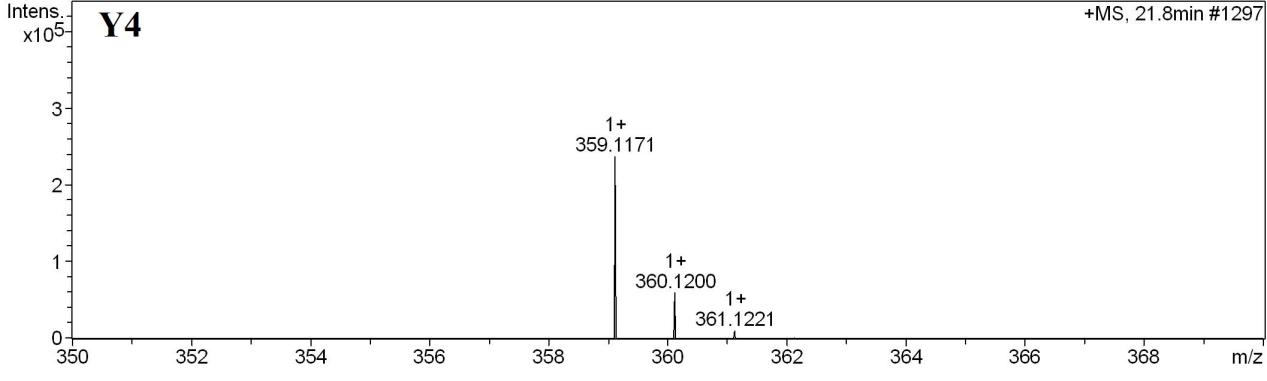


**Fig.S3** LC-MS analysis of extracellular pigments. **a** Total ion chromatograms, absorption traces of the pigments. **Y1-Y4**, Mass spectra and their collision-induced fragmented data.
